# Draft Genome Sequence of *Elizabethkingia anophelis* Strain EM361-97 Isolated from the Blood of a Cancer Patient

**DOI:** 10.1128/genomeA.01215-16

**Published:** 2016-10-27

**Authors:** Jiun-Nong Lin, Chih-Hui Yang, Chung-Hsu Lai, Yi-Han Huang, Hsi-Hsun Lin

**Affiliations:** a Department of Critical Care Medicine, E-Da Hospital, I-Shou University, Kaohsiung, Taiwan; b Division of Infectious Diseases, Department of Internal Medicine, E-Da Hospital, I-Shou University, Kaohsiung, Taiwan; c College of Medicine, I-Shou University, Kaohsiung, Taiwan; d Department of Biological Science and Technology, Meiho University, Pingtung, Taiwan

## Abstract

*Elizabethkingia anophelis* EM361-97 was isolated from the blood of a patient with nasopharyngeal carcinoma and lung cancer. We report the draft genome sequence of EM361-97, which contains a G+C content of 35.7% and 3,611 candidate protein-encoding genes.

## GENOME ANNOUNCEMENT

*Elizabethkingia*, previously belonging to genus *Flavobacterium* and then *Chryseobacterium*, is a Gram-negative, nonfermentative rod that is ubiquitously distributed in soil, water, and reservoirs (1). This microorganism has rarely been reported to cause diseases in humans before. However, *Elizabethkingia* has recently emerged as an important pathogen in the opportunistic infections of immunocompromised patients and neonates. The most common infections of *Elizabethkingia* include pneumonia, bacteremia, meningitis, and neutropenic fever (2–4). The genus *Elizabethkingia* includes four species: *E. meningoseptica*, *E. miricola*, *E. anophelis*, and *E. endophytica* (5). *E. anophelis*, first isolated from the mosquito *Anopheles gambiae* in 2011, has caused several outbreaks of infections in the United States (3) and Hong Kong (4). Infections of *E. anophelis* are associated with a mortality rate of 24% to 30% in humans (3, 4).

*E. anophelis* strain EM361-97 was isolated from the blood of a patient with advanced nasopharyngeal carcinoma and lung cancer in Taiwan. This patient has received several courses of radiotherapy and chemotherapy. This isolate was identified as *E. anophelis* according to the results of 16S rRNA gene sequencing (6).

Total DNA of the isolate was prepared using a Wizard genomic DNA purification kit according to the manufacturer’s instructions (Promega, WI, USA). The genomic DNA was sequenced using an Illumina HiSeq 2000 sequencing platform (Illumina, CA, USA). A total of 1,463 Mb data was produced and the short reads were assembled into a genome sequence using the SOAP *de novo* method (7). The total length of the draft genome was 4,077,699 bp with a mean G+C content of 35.7%. The assembly contained 26 scaffolds and 27 contigs. Gene prediction was performed by the NCBI Prokaryotic Genome Annotation Pipeline (8). The methods of best-placed reference protein set and GeneMarkS+ were used for the annotation of genes, coding sequences (CDSs), rRNAs, tRNAs, noncoding RNAs (ncRNAs), and repeat regions (8). A total of 3,738 genes and 3,663 CDSs were identified. The total length of genes makes up approximately 87.9% of genome. There were 52 pseudo genes. The predicted number of coding genes was 3,611. The number of RNA genes was 75, including 21 rRNAs (5S: 5; 16S: 7; 23S: 9), 51 tRNAs, and three ncRNAs.

Among the species of genus *Elizabethkingia*, *E. meningosepticum* is the most well-known species (9–11). In contrast, less information is available about the epidemiology, virulence factors, antibiotics resistance, and clinical manifestations of *E. anophelis*. Knowledge of the genome sequence of *E. anophelis* will provide researchers important information to understand the pathogenicity of this emerging microorganism.

### Accession number(s).

This whole-genome shotgun project has been deposited at GenBank under the accession number LWDS00000000.
